# Cajal’s legacy in the digital era: from neuroscience foundations to deep learning

**DOI:** 10.3389/fnana.2025.1672016

**Published:** 2025-10-15

**Authors:** Marcos García-Lorenzo, Oscar Herreras, Javier DeFelipe

**Affiliations:** ^1^VG-LAB, Universidad Rey Juan Carlos, Móstoles, Spain; ^2^Instituto Cajal, CSIC, Avenida Doctor Arce, Madrid, Spain; ^3^Laboratorio Cajal de Circuitos Corticales, Centro de Tecnología Biomédica, Universidad Politécnica de Madrid, Madrid, Spain

**Keywords:** brain circuits, neuron theory, theory of dynamic polarization, neural networks, artificial intelligence, deep learning

## Abstract

Santiago Ramón y Cajal’s pioneering work laid the foundations for modern neuroscience and continues to impact the development of artificial intelligence, particularly deep learning. His neuron theory, the principle of dynamic polarization, and his insights into brain plasticity and network organization have significantly influenced both our understanding of the nervous system and the design of artificial neural networks. This article reviews Cajal’s key contributions, explores their role in the evolution of AI, and emphasizes the enduring links between neuroscience and machine learning in the digital era.

## 1 Introduction

Groundbreaking research into the structure and function of the nervous system by Santiago Ramón y Cajal (1852/1934) marked a pivotal turning point in neuroscience and earned the recognition as the father of the field (DeFelipe, 2002). Cajal’s early work depended on a powerful new technique, the “black reaction” (reazione nera), developed by Camillo Golgi in 1873. This innovation revolutionized the study of the nervous system’s anatomy by allowing, for the first time, nerve cells to be seen in remarkable detail—including their cell body, dendrites, and axon. In honor of its creator, this staining technique became known as the Golgi method. At that time, the prevailing theory about nervous system organization was the reticular theory, which proposed that the elements of the nervous system formed a continuous, interconnected network. Ironically, despite the clarity produced by his own method, Golgi himself was the primary advocate of this theory, suggesting that while dendrites ended freely, axonal branches interconnected to create an extensive “rete nervosa diffusa” (diffuse nervous network). A few years later, Cajal adopted Golgi’s method and in 1888 published his first major work using this technique, titled “Estructura de los Centros Nerviosos de las Aves” (“Structure of the Nerve Centers of Birds”). In this article, he described for the first time the presence of small protrusions on the dendrites of certain nerve cells, which he called “dendritic spines”—structures that remain a focus of research today. Furthermore, while confirming Golgi’s conclusion that dendrites end freely, Cajal also added the decisive observation that the same applies to axons and their branches. This “free” (without anastomosis) and “varicose” (with axonal dilations) arborization led Cajal to declare that “each element [nerve cell] is a physiologically absolutely autonomous canton.” This observation represented a radical shift in the understanding of brain function, moving from the idea of a continuous neural network to that of an “infinitely fragmented” brain. This raised the critical question of how the nerve impulse is transmitted from one nerve cell to another across a physical gap. Cajal continued to collect extensive evidence supporting neuron theory across many regions of the nervous system and in different species. Between his initial use of the Golgi method in 1887 and his formal statement of the Neuron Doctrine in 1891, Cajal published more than 30 articles. The strength of these studies formed the foundation of a classic and influential review in 1891 by Waldeyer-Hartz, who officially introduced the term “neuron” for the nerve cell.

Cajal’s contributions—including the neuron theory, the principle of dynamic polarization, his ideas on brain plasticity as the basis for memory and learning, and the concept of avalanche conduction (in which a single stimulus can activate many neurons across multiple brain regions)—represent a profound legacy that endures into the digital era. These concepts were published in numerous articles and summarized in his seminal book, Textura del sistema nervioso del hombre y de los vertebrados (1899–1904), which was later more widely disseminated in a French edition by Leon Azoulay that included additional texts and figures (Cajal, 1909/1911). The following section describes how these ideas have inspired artificial neural networks.

## 2 Cajal’s foundational concepts in neuroscience

Cajal’s neuron theory, which established that neurons are individual cells that communicate through synapses, represents a foundational principle in neuroscience that has significantly influenced the development of artificial intelligence (AI). As Cajal stated (Cajal, 1889):

We have never been able to see a mesh of such a network [Golgi’s axonal network], neither in the cerebrum, nor in the spinal cord, nor in the cerebellum, nor in the retina, nor in the olfactory bulb, etc. We believe that it is time now to disengage histology from any physiological commitment, and simply adopt the only opinion that is in harmony with the facts, namely: that nerve cells are independent elements never anastomosed either by their protoplasmic expansions [dendrites] or by the branches of their Deiters’ prolongation [axon], and that the propagation of nerve action occurs by contacts at the level of certain apparatuses or gearing arrangements, whose purpose is to fix the connection.

**Figure 1:**
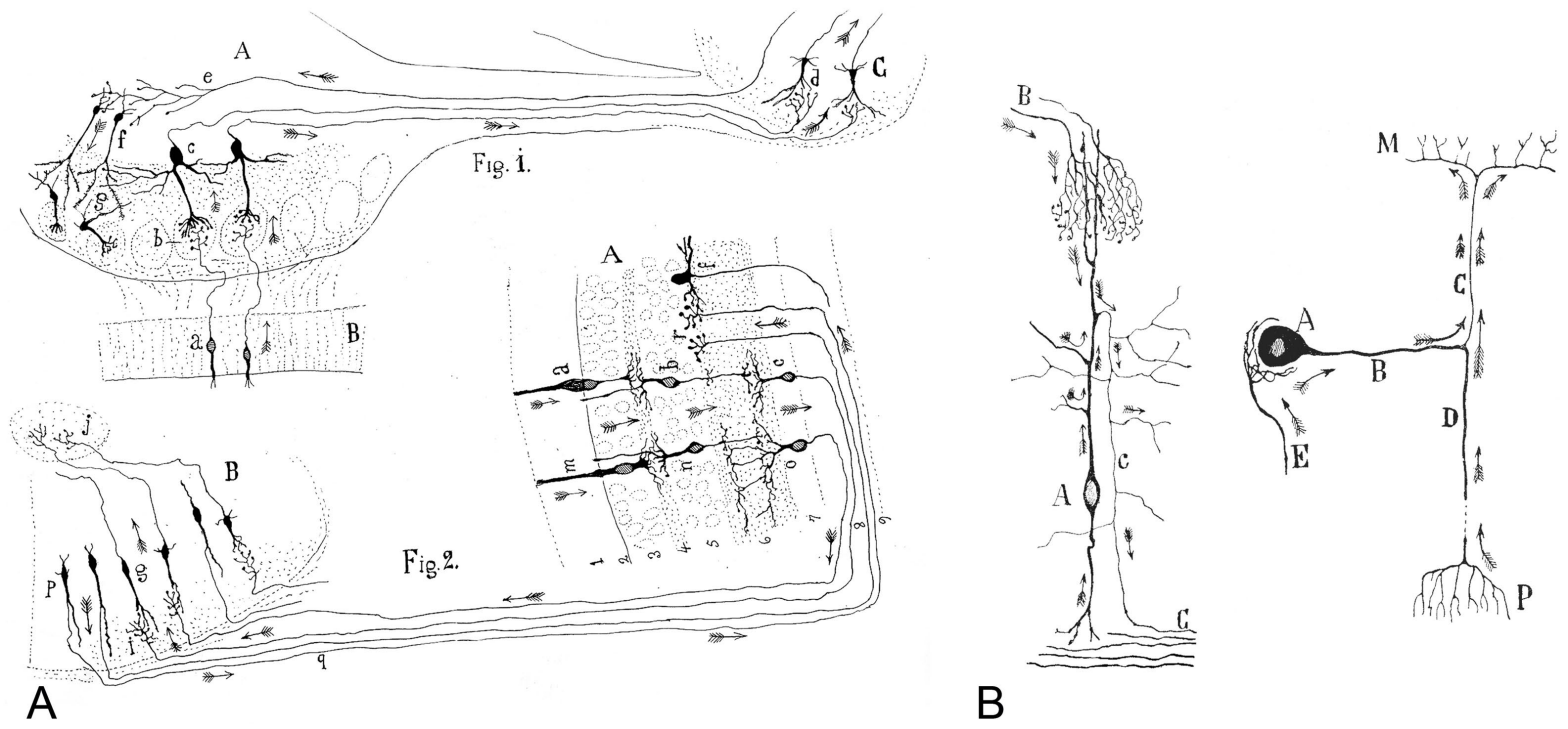
**(A)** Cajal’s scheme showing the current flow in the visual and olfactory systems. This drawing was reproduced in his article *Significación fisiológica de las expansiones protoplásmicas y nerviosas de las células de la substancia gris* (Cajal, 1891). The legend states: “Fig. 1. Scheme of cellular connections in the olfactory mucosa, olfactory bulb, tractus, and olfactory lobe of the cerebrum. The arrows indicate the direction of the currents. **A**, olfactory bulb; **B**, mucosa; **C**, olfactory lobe. **a**, **b**, **c**, **d**, one-way or centripetal pathway through which sensory or olfactory excitation passes. **e**, **f**, **g**, centrifugal pathway through which the [nervous] centers can act on the elements of the bulb, granules and nerve cells, whose protoplasmic processes penetrate the glomeruli. Fig. 2. Scheme of the visual excitation pathway through the retina, optic nerve and optic lobe of birds. **A**, retina; **B**, optic lobe. **a**, **b**, **c**, represent a cone, a bipolar cell and a ganglion cell of the retina, respectively, the order through which visual excitation travels. **m**, **n**, **o**, parallel current emanating from the rod also involves bipolar and ganglion cells. **g**, cells of the optic lobe that receive the visual excitation and transfer it to **j**, the central ganglion. **p**, **q**, **r**, centrifugal currents that start in certain fusiform cells of the optic lobe and terminate in *r*, in the retina at the level of the spongioblasts; **f**, a spongioblast. Arrows indicate the direction of current flow”. **(B)** The directional flow of the nervous current within the neuron. Cajal’s drawing reproduced in his article *Leyes de la morfología y dinamismo de las células nerviosas* (Cajal, 1897). The legend states: Left, “Crosier cell of the optic lobe of the sparrow. **A**, soma; **B**, fibers arriving from the retina; **c**, central white matter; **C**, axon. Arrows indicate the direction of the current [flow].” Right, “Scheme showing the current flows in a sensory ganglion cell of mammals. **A**, soma; **B**, shaft; **D**, axipetal or peripheral process that provides currents; **C**, axon that carries the impulses to the spinal cord; **E**, fiber constituent of the pericellular arborization; **M**, spinal cord; [P, skin]”.

A major outcome of Cajal’s neuron theory was the formulation of the Law of Dynamic Polarization in nerve cells. At the time, the mechanisms by which nerve impulses traveled within neurons were largely unknown. The prevailing belief was that dendrites served a primarily nourishing function, while axons transmitted nerve impulses away from the cell body (cellulifugal direction)—a notion mainly derived from observations of axonal conduction in spinal motor neurons. Nevertheless, the specific role of dendrites in processing information remained unclear, and there was no widespread agreement or definitive explanation at the time (DeFelipe, 2018). Cajal (1889) believed it was clear that the dendrites played a role in receiving currents, at least in certain cases, and 2 years later he tried to generalize this idea in the Law of Dynamic Polarization (Cajal, 1891). Cajal inferred the principle of impulse directionality by studying systems such as the visual and olfactory pathways, where the flow of neural signals was particularly clear (Figure 1A):

If in such inquiry, the [dendritic] arborization is always shown as a receptor apparatus and the [axonal arborization] as an apparatus for the application of the [impulses], then by analogy we would have attained a rule to judge the direction of the currents in the [nerve cells within the central nervous system].

Cajal proposed that neurons could be divided into three functionally distinct regions: a receptor apparatus (formed by the dendrites and soma), an emission apparatus (the axon) and a distribution apparatus (terminal axonal arborization). He later realized that the soma does not always intervene in the conduction of the impulses and that sometimes impulse activity goes directly from the dendrites to the axon (Figure 1B; Cajal, 1897). Thus, the law of dynamic polarization became the theory of axipetal polarization:

The soma and dendrites display axipetal conduction, whereby they transmit the nervous waves towards the axon. Conversely, the axon or cylinder-axis has somatofugal or dendrifugal conduction, propagating the impulses received by the soma or dendrites towards the terminal axonal arborizations. […]. This formula can be applied universally without exception, both in vertebrates and invertebrates.

**Figure 2:**
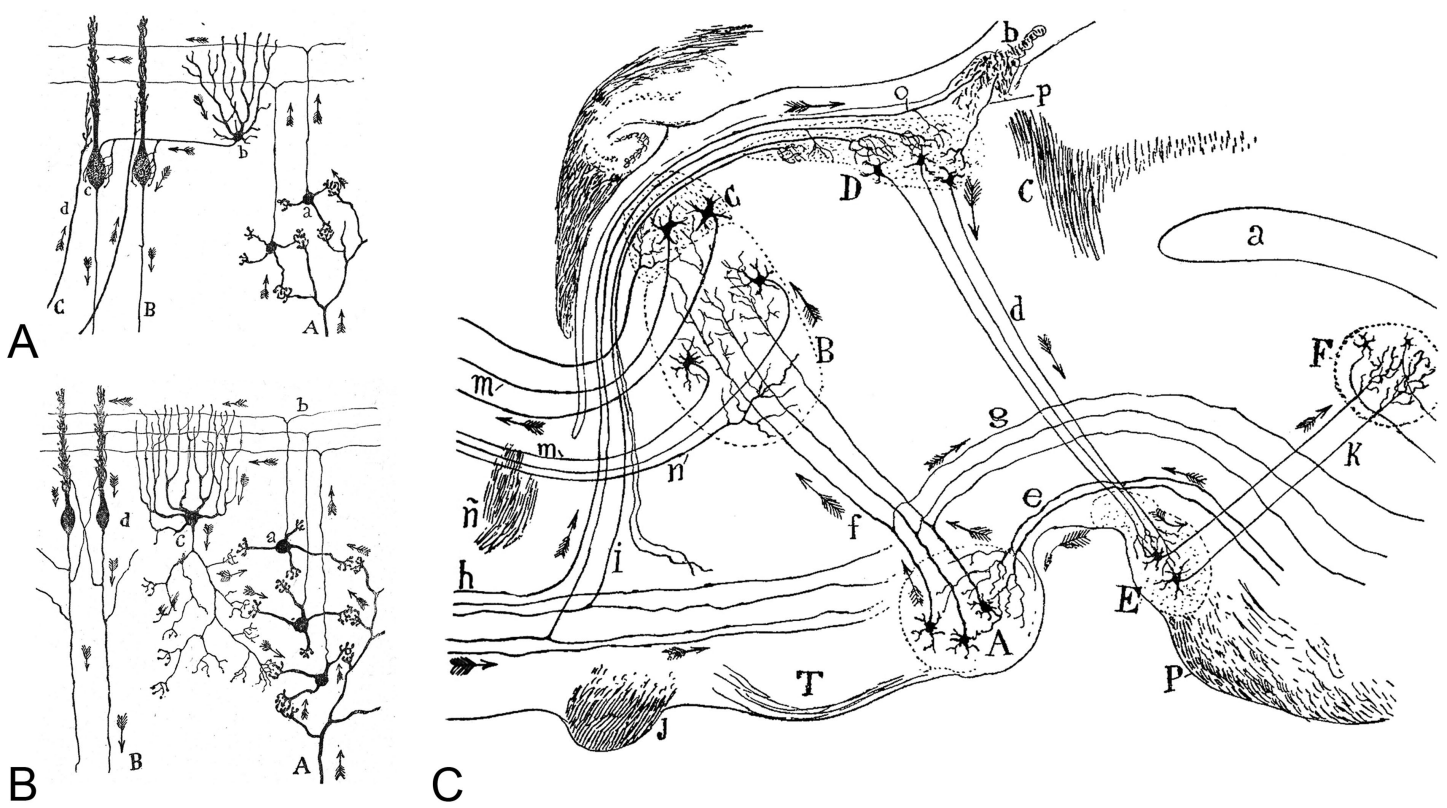
**(A,B)** Cajal’s drawing to illustrate the participation of different types of cells in the transmission of impulses in the cerebellum based on the theory of dynamic polarization. **A**, The legend states: “Diagram that reveals the flow of the current contributed by the mossy fibers and the role of the Golgi cells in it. **A**, mossy fibers; **B**, Purkinje cell axons; **a**, granule cells; **b**, parallel fibers; **c**, Golgi cell; **d**, side view of Purkinje cell”. Taken from Cajal (1899/1904). **B**, The legend states: “Diagram destined to show the participation of basket cells in the transmission of the afferent impulses. **A**, mossy fiber; **B**, Purkinje cell axons; **C**, climbing fiber; **a**, granule cells; **b**, basket cell; **c**, [side view of] Purkinje cell.” Taken from Cajal (1899/1904). **(C)** Diagram of the afferent and efferent pathways of the mammillary body, habenular nuclei, and anterior and medial thalamic nuclei. The legend states: “**A**, medial mammillary nucleus; **B**, anterior medial nucleus of the thalamus; **C**, anterior ventral nucleus; **D**, habenular nuclei; **E**, interpeduncular nucleus; **F**, dorsal tegmental nucleus; **J**, optic chiasm; **K**, tegmental bundle of the interpeduncular nucleus; **T**, tuber cinereum; **P**, pons; **a**, cerebral aqueduct of Sylvius; **b**, habenular commissure; **c**, posterior commissure; **d**, fasciculus retroflexus (or Meynert’s fasciculus); **e**, peduncle of the mammillary body; **f**, mammillothalamic tract (or Vicq d’Azyr’s bundle); **g**, mammillotegmental tract (or Gudden’s tegmental fasciculus); **h**, stria terminalis; **i**, stria medullaris; **m**, thalamocortical fibers; **n**, corticothalamic fibers; **o**, fiber of the stria medullaris traveling to the habenular commissure to arborize within habenular nuclei of the contralateral side; ñ, anterior commissure; **p**, fiber originating from the opposite side. The arrows indicate the direction of the currents. Taken from Cajal (1903).

Cajal argued that if dendrites consistently function as receptive structures and axons as transmitting elements, this organizational model could be applied broadly to determine the direction of impulse transmission across the central nervous system, regardless of neuron type or brain region, as depicted in Figures 1, 2. As we will discuss below, Cajal’s theory of dynamic polarization was fundamental in mapping and understanding how information flows through the brain’s complex microcircuits. This principle enabled Cajal and his contemporaries to determine and interpret the direction of information flow within the intricate networks of the nervous system.

However, the concept of inhibition as a neurophysiological process that reduces the activity of other neurons was absent from Cajal’s description of network function. Our current understanding of inhibitory mechanisms emerged later, following John Eccles’ demonstration of chemical synaptic transmission (Eccles, 1961). As discussed in Herreras (2025), during the 1930s and 1940s single cell recordings were common, and the first intracellular recordings were made. These studies were critical in forging many of the basic concepts of neuronal physiology that conformed to the doctrine of dynamic polarization—namely, the concept of spatiotemporal integration of excitatory and inhibitory synaptic currents, the electrotonic (passive) propagation of synaptic currents within dendrites (i.e., with pronounced decay), and the attribution of the role of spike trigger zone to the axon initial segment (AIS) (Lorente de Nó, 1935; Eccles, 1957; Rall, 1967). Thus, dendrites were assigned the role of a synaptic input zone and the neuron was globally considered a passive integrator of inputs.

In many of Cajal’s illustrations, he mapped how axons from different functional regions of the brain connect with neurons in specific areas, demonstrating the integration of inputs from diverse sources (Figure 2C). He also depicted how these neurons then distribute the ‘processed’ information both within the region where they are located and to other parts of the brain. Furthermore, Cajal’s theories, along with his ideas on plasticity and hierarchical processing of brain circuits, have provided biological inspiration for key concepts in artificial neural networks, which are fundamental to AI: (i) The concept that neurons transmit signals in a structured network serves as a fundamental principle in designing artificial neurons in AI models; (ii) Cajal’s observations suggesting that neural structures change with experience have parallels in AI learning algorithms, particularly in adaptive and self-improving systems; and (iii) his descriptions of how different brain regions process information in layers influenced deep learning (DL) architectures, where multiple layers of artificial neurons process data hierarchically.

## 3 Rafael Lorente de Nó and the concept of reverberating circuits

**Figure 3:**
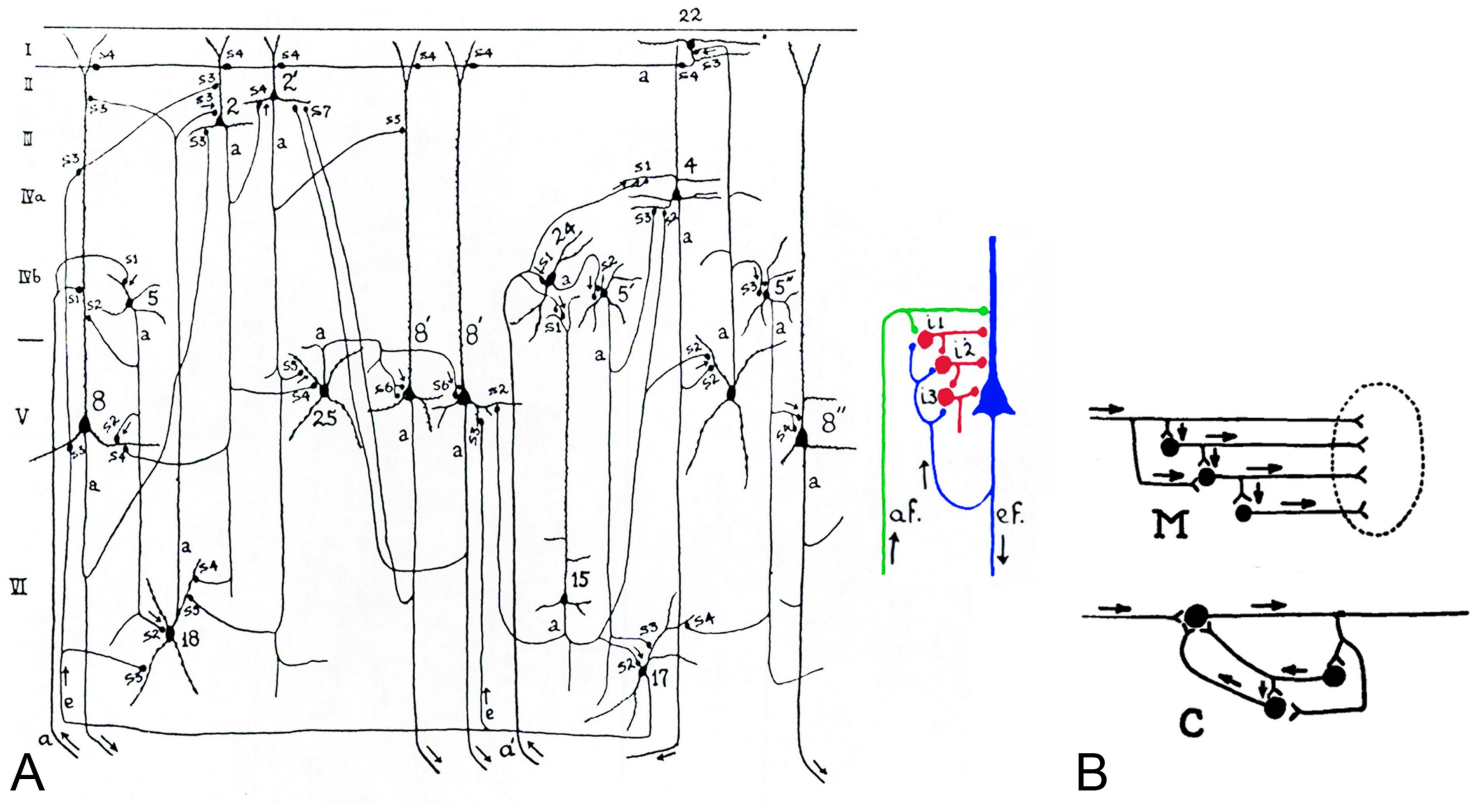
**(A)** Diagram of some of the intracortical chains of neurons taken from Lorente de Nó (1938b). The legend states: “The number on the cells and the letters a and e on the fibers are the same as in figures 63, 64 and 65. The axons of the cortical cells are marked with a. Note that only a few dendrites and axonal branches have been included in the diagram. The synaptic junctions are indicated with the letter s (s1, s2, etc.) and with a thickening of the axon. It is assumed that the synapses marked with an arrow are passed by the impulses. The small diagram at the right is a simplification of the diagram at the left. The afferent fiber af. activates the large pyramid which is the origin of an efferent fiber ef. and also a system of cortical internuncial cells (i1, i2, i3); the recurrent collateral of ef delivers impulses again to the internuncial system. This diagram summarizes the plan upon which the central nervous system is built.” The small diagram has been colored by the authors of the present work for greater clarity. **(B)** Lorente de Nó’s diagrams to illustrate the two types of chains formed by internuncial cells that he distinguished between—**M**, multiple and **C**, closed chain. Modified from Lorente de Nó (1938a).

Another early and significant contribution to this field was made by Lorente de Nó (1902/1990), one of Cajal’s most distinguished disciples, who in 1938 described in greater detail than previous authors the direction of impulse transmission in the cerebral cortex based on Cajal’s law of dynamic polarization, and introduced the novel and key concept of local reverberating circuits (Lorente de Nó, 1938a; Lorente de Nó, 1938b; Figure 3).

As outlined in Herreras (2025), this concept of reverberating circuits laid the foundation for contemporary perspectives regarding brain function, with profound implications extending well beyond neuroscience. Lorente’s exchange of ideas on looping circuits with Norbert Wiener and his contemporaries gave rise to cybernetics and control theory (Wiener, 2019) and also anticipated Hebb’s concept of cell assembly (Espinosa-Sánchez et al., 2019). The concept of recurrent circuits introduced a fundamental principle in neuroscience: feedback. This notion marked a decisive departure from the classical reflex arc paradigm, wherein neural responses were viewed as immediate, automatic, and unidirectional. By contrast, recurrent circuits, characterized by reciprocal neuronal connections that form closed-loop pathways, allow signals to cycle back within a network. This reverberatory activity enables neural responses to be modulated, sustained, or stored, thereby providing the substrate for complex cognitive processes. Functions such as working memory maintenance, temporal sequence processing, feedback regulation, sustained attention, and decision-making critically depend on these dynamic recurrent interactions.

## 4 Definition of artificial intelligence

Although Artificial Intelligence (AI) has shown great potential for solving problems across various fields and disciplines throughout its nearly 70-year history, it is the advances of recent decades that have brought this technology closer to society and made it more widely used. The widespread enthusiasm for AI’s achievements is not always matched by a clear understanding of the technology and its scope. The definitions offered by relevant organizations and the scientific community are often vague and disconnected from the reality of the field. AI is frequently described as a technology that enables machines and computers to simulate or display human intelligence—an idea closely tied to the original concept of the field. Today, the body of knowledge is so broad and diverse that it is difficult to define AI precisely without excluding certain technologies that fall within its scope. On the other hand, making the definition too general would blur the lines with traditional computing. Evidence of this difficulty can be found in the multiple attempts made by the European Commission to establish and refine a definition (European Commission: High-Level Expert Group on Artificial Intelligence, 2018; Samoili et al., 2020; Regulation (EU), 2024/1689; European Commission, 2025). In its first attempt to delineate both the academic field and the technological scope, the European Commission: High-Level Expert Group on Artificial Intelligence (2018) requested a report from a group of experts from academia and industry. This group drew on the proposal by Russell and Norvig (2009), which replaces the concept of intelligence with that of rationality.

Artificial intelligence (AI) systems are software (and possibly also hardware) systems designed by humans […] that, given a complex goal, act in the physical or digital dimension by perceiving their environment through data acquisition, interpreting the collected structured or unstructured data, reasoning on the knowledge, or processing the information, derived from this data and deciding the best action(s) to take to achieve the given goal. AI systems can either use symbolic rules or learn a numeric model, and they can also adapt their behavior by analysing how the environment is affected by their previous actions. As a scientific discipline, AI includes several approaches and techniques, such as machine learning […], machine reasoning […], and robotics […].

This definition of AI highlights two key aspects: the broad scope of the field and the collective effort by technicians and scientists to move away from the traditional concept of human intelligence. The attempt to redefine intelligence in terms of rationality and efficiency reflects a clear focus on problem-solving capabilities, setting aside the historical associations with biological intelligence. While artificial intelligence was originally closely linked to neuroscience, the boundaries between the two disciplines are now clearly defined. The exponential growth and increasing specialization of each area have led many AI researchers to focus exclusively on algorithmic and mathematical methods, leaving aside contributions from the study of the brain (except for the field of neuromorphic computing). This separation, although understandable in the context of rapid evolution, raises questions about the potential loss of synergies that could enrich both fields, as was highlighted in an excellent article by Hassabis et al. (2017).

One of the most prolific areas of artificial intelligence today is machine learning (ML), a discipline whose goal is to enable algorithms to learn functions that approximate solutions to complex problems based on data. ML techniques mainly differ in the type of functions they can automatically learn from data (Alpaydin, 2020). Within this wide range of methods, the most promising techniques at present are those based on artificial neural networks (ANN), regarded by many as potential candidates for achieving artificial general intelligence (AGI) and artificial superintelligence (ASI). AGI refers to systems capable of performing any cognitive task at a human level, going beyond the narrow specialization of current models (Goertzel, 2014). ASI describes systems that would surpass human cognitive abilities across virtually all domains (Bostrom, 2014). ANNs take inspiration from the organization of the human brain and rely on the idea that the coordinated action of simple units—nodes or “neurons”—can produce complex emergent behavior (Russell and Norvig, 2009). The early conceptualization of brain circuits proposed by Cajal and Lorente de Nó changed the understanding of brain function and introduced principles that later served as a foundation for ANN design. The principle of dynamic polarization supports directed connectivity and layered organization in artificial models. Lorente de Nó’s description of reverberating circuits provided the basis for classifying ANNs into two groups: feedforward and recurrent architectures. In feedforward networks, information moves in one direction from input to output, which suits tasks without temporal dependence; examples include multilayer perceptrons (MLPs; Rumelhart et al., 1986), autoencoders (Hinton and Salakhutdinov, 2006), and convolutional neural networks (CNNs; LeCun et al., 1998). In recurrent neural networks (RNNs; Hopfield, 1982), feedback connections create an internal state and enable the processing of sequences, supporting the modeling of time and memory; examples include vanilla RNNs, long short-term memory networks (LSTMs; Hochreiter and Schmidhuber, 1997), and gated recurrent units (GRUs; Cho et al., 2014). It should be noted that not all architectures fit within this scheme; Boltzmann machines (Ackley et al., 1985) and self-organizing maps (Kohonen, 1982) are notable examples that are both designed to find hidden patterns in data without human supervision. Building a complete and consistent taxonomy of neural models remains a difficult task and is beyond the scope of this article (Samoili et al., 2020; Sarker, 2021). The concept of neural plasticity also influenced early progress in training methods. The idea of neural plasticity also influenced early approaches to training, suggesting that connection strengths could be modified through learning. These principles continue to shape contemporary models in neuroscience and in artificial intelligence. Nevertheless, although present in the formative period of the field, the contributions of Cajal and Lorente de Nó were not made explicit in the first publications on ANNs. The aim of this article is to exemplify these early contributions and to show how their underlying ideas remain relevant today. For a comprehensive historical survey of the field, readers are referred to the overview by Schmidhuber (2015).

## 5 From biological networks to AI

Although pioneering works that laid the foundations of modern AI already existed—such as Turing (1950) paper, which discussed the possibility that machines could think, or McCulloch and Pitts’s (1943) study, which introduced the first mathematical model of neurons and established a theoretical framework for neural processing—it is generally agreed that the 1956 Dartmouth workshop marked the beginning of modern research in this area. No formal proceedings were published at that event; however, the original proposal (McCarthy et al., 1955) by the four organizers—John McCarthy, Marvin Minsky, Nathaniel Rochester, and Claude Shannon—is still available. This proposal outlined lines of research that remain valid to this day. It is striking to see how, at such an early stage of the technology, this project defined areas that would later prove fundamental to the field: complexity and computation theory; search algorithms; the study of natural language; self-improvement and machine learning; creativity and randomness; and artificial neural networks. Right from the opening paragraph, the text emphasizes the need to understand human intelligence as a reference for the development of thinking machines:

The study is to proceed on the basis of the conjecture that every aspect of learning or any other feature of intelligence can in principle be so precisely described that a machine can be made to simulate it.

Among the four research lines described in the Dartmouth proposal, the analysis of the neuron, the study of its interrelationships, and the exploration of the human brain were core pillars. From the outset, it was assumed that the simulation of neural networks could generate, from simple interactions, emergent behaviors that might be considered intelligent. Several works prior to the Dartmouth workshop laid the foundation for progress in this direction.

The aforementioned study by McCulloch and Pitts (1943) presents a first formulation of neuronal activity and neural networks in terms of propositional logic. The authors argue that the “all-or-none” nature of nerve activity allows neuronal events and their relationships to be modeled using logic. Although not mentioned explicitly, this approach builds on Cajal’s view of the nervous system as a network of discrete elements, in which the neuron serves as the logical unit of that network. The essential features Cajal described—a soma receiving signals from prior synapses, and an axon transmitting impulses—are reflected in McCulloch and Pitts’s framework. Essentially, their theory assumes that each neuron’s activity can be represented as a logical proposition, and that the physiological relationships between these activities correspond to relationships between these propositions. The article further introduces concepts such as the excitation threshold, the latent addition period, and the synaptic delay, translated into logical terms.

In his doctoral dissertation (Minsky, 1954), Marvin Minsky—one of the Dartmouth conference organizers—continued the legacy of Cajal’s neuronal theory. His work explored analog neural networks, incorporating temporal quantization and generalizing the notion of the neuron through the idea of the *cell*. Information flows according to dynamic polarization, so that a cell’s state at time t depends on the state of preceding cells at the previous instant ( t−1 ). Although this neural network model was and remains one of his main contributions, the core goal was to propose a new approach to studying processes such as learning, memory, recognition, and attention. Minsky also describes a reinforcement operator that relates closely to neuronal plasticity and that considers the influence of past states on the system’s future behavior. This reflects how Cajal’s observations—namely, that neuronal structures change with experience—are manifested in adaptation and learning mechanisms (DeFelipe, 2006).

**Figure 4:**
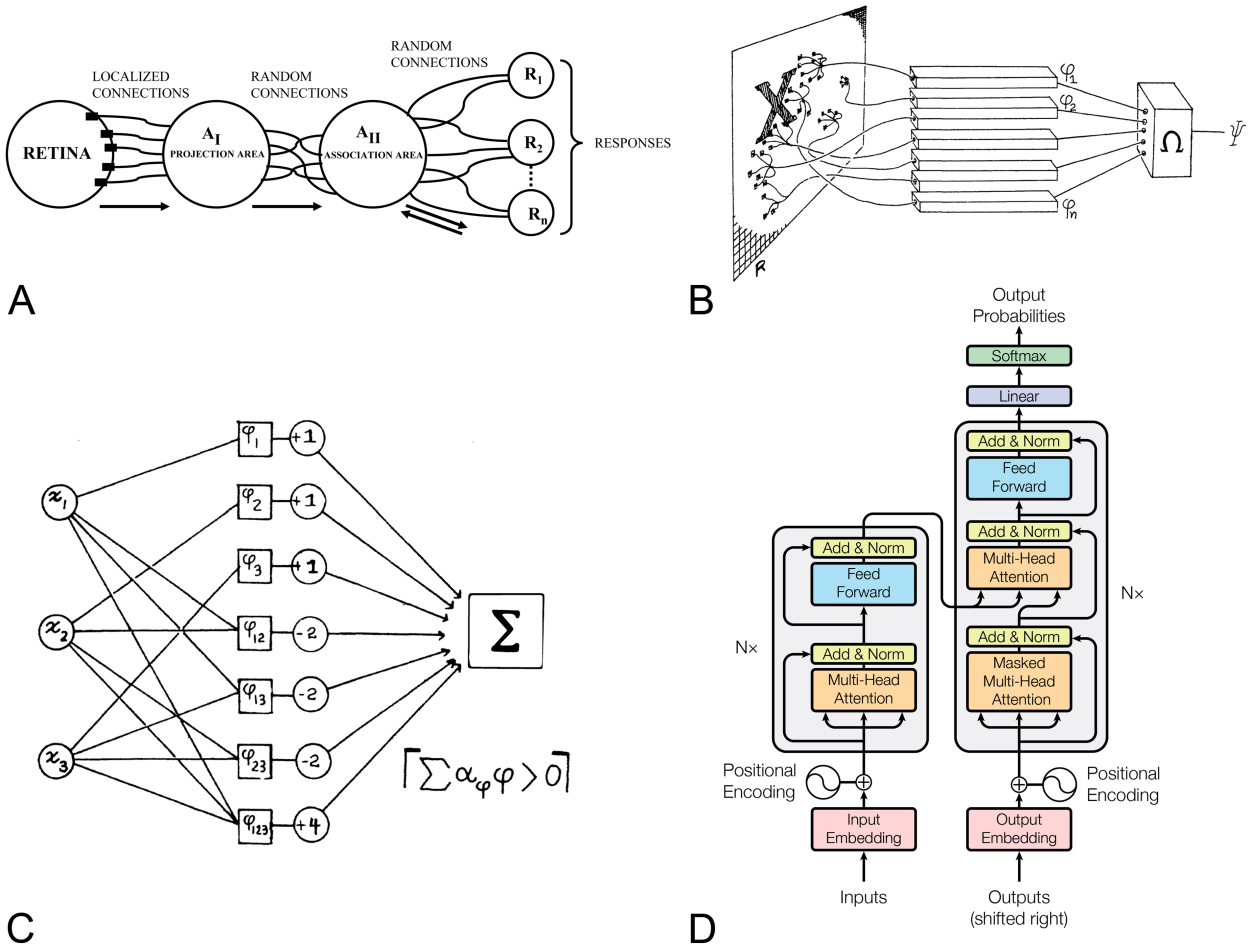
The perceptron across three generations of neural models. Together, panels **(A–D)** show how the perceptron—conceptually rooted in Cajal’s anatomical description of the neuron and his claim that learning materializes as circuit plasticity—remains a fundamental computational element, from early neurobiological analogies to present-day transformer models. **(A)** Schematic of the perceptron architecture adapted from Rosenblatt (1958). Stimulus-receiving S -cells (Retina) activate projection-area association cells ( $A_{I}$ ), which in turn excite higher-order association cells ( $A_{\mathrm{II}}$ ); bidirectional synapses connect $A_{\mathrm{II}}$ to response cells ( R -units). Learning proceeds through the formation and adjustment of these synapses, embodying the connectionist hypothesis that memory is stored in connections. **(B,C)** Feed-forward linear-threshold formulation analyzed by Minsky and Papert (1969). A predicate ψ(X) takes the value 1 when the weighted sum of basic predicates φ(X) exceeds a fixed threshold θ , i.e., $\;\psi (X)=[\sum \alpha_{\varphi }\varphi (X)>\theta ].$ Removal of feedback highlights the perceptron as a device that realises linear decision boundaries and exposes its logical limitations. **(D)** Encoder–decoder architecture introduced in *Attention Is All You Need* (Vaswani et al., 2017). Each transformer block contains a position-wise multi-layer perceptron that follows the self-attention sub-layer, illustrating the perceptron’s role as the basic feed forward unit in contemporary deep-learning stacks. **(A)** Reproduced from Rosenblatt (1958). Reprinted with permission from the American Psychological Association. © 1958 American Psychological Association. **(B,C)** Reproduced from Minsky and Papert (1969). Reprinted with permission from MIT Press. © 1969 Massachusetts Institute of Technology. **(D)** Reproduced from Vaswani et al. (2017). Used under permission granted by Google for journalistic and scholarly works. © 2017 Google LLC.

Later, in 1958, Frank Rosenblatt introduced the perceptron as a probabilistic learning model, marking a decisive break with the logical, rule-based networks of McCulloch and Pitts (1943) and the early work of Minsky (1954). Although Rosenblatt did not cite either Cajal or Lorente de Nó, their influence is evident in his design. The photo-perceptron (Figure 4A) mirrored Cajal’s concept of layered processing (Figure 1) and differentiated cell types (Figure 2) by organizing sensory (S-points), association (A-units) and response (R-units) elements. In this framework, Cajal’s principle of dynamic polarization—information flowing from dendrite to axon—was recast as adjustable synaptic weights that embody neural plasticity (Rosenblatt, 1958). Echoing Lorente de Nó’s idea of reverberating circuits, Rosenblatt incorporated bidirectional connections between A- and R-units, adding feedback loops, potential inhibitory signals and an early glimpse of recurrence. The perceptron was more than a theoretical construct: Rosenblatt implemented it on an IBM 704 and subsequently built the Mark I Perceptron, demonstrating that biologically inspired learning algorithms could be embodied in hardware, revealing the true potential of this approach.

The feed-forward version examined in Perceptrons (Minsky and Papert, 1969) removed Rosenblatt’s feedback connections, simplifying the original model and bringing it closer to today’s definition of a perceptron. This restriction formalized linear classification theory and provided the mathematical basis for the first supervised-learning algorithms. At the same time, the analysis exposed the severe performance limits of single-layer classifiers and temporarily slowed progress in the field. These limitations were overcome with multi-layer networks and the back-propagation algorithm (Rumelhart et al., 1986), which tunes synaptic weights in a manner consistent with Cajal’s plasticity. Meanwhile, the feedback present in Rosenblatt’s original design inspired later developments in recurrent networks and associative memories (Hopfield, 1982). Today, multi-layer perceptrons remain the fundamental building block of advanced models—including transformer-based systems (Vaswani et al., 2017) shown in Figure 4—highlighting how Rosenblatt’s probabilistic, biologically grounded perspective continues to shape modern DL.

Another interesting point to consider is Cajal’s vision of the functional organization of neuronal networks when he addressed the question of whether each perception has one or more nerve cells as its substrate (Cajal, 1895). To explain this, he proposed what he called “avalanche conduction.” In Cajal’s own words:

Research in recent years on the structure of the nervous system has revealed that between the sense organs and the nerve centers there is a fixed chain of conductors or neurons, in which the impression received in the periphery by a single sensory cell is propagated in an avalanche—that is, by an increasing number of cells—to the brain.

In other words, simple units (neurons) can generate increasingly complex emergent behavior as the network size grows. Cajal’s avalanche conduction concept laid the groundwork for our modern understanding that both biological and artificial neural networks achieve complex, emergent behaviors through the collective activity of many simple units. This aligns with numerous studies confirming that artificial neural networks improve their performance as the number of layers increases (Hornik et al., 1989; Kidger and Lyons, 2020). In this regard, the introduction of the term *deep learning* and pretrained deep neural networks (Hinton et al., 2006) marked a milestone in the designing of more capable models. Among these architectures, *transformers* currently stand out due to their significant scalability. While training them requires large amounts of data, there are no clear limits to their potential when network size and training data volume increase (Cordonnier et al., 2020; Ravfogel et al., 2019). Many studies have analyzed their scaling properties (Hoffmann et al., 2022; Kaplan et al., 2020; Pearce and Song, 2024). Today, models with very large numbers of parameters are being trained—for example, Llama 4, with over one trillion parameters^1^—on massive and diverse datasets, resulting in what are known as “foundation models” (Bommasani et al., 2021). These models can be reused and adapted to tasks beyond their original training sets (Brown et al., 2020; Liu et al., 2019). Although they originated in natural language processing (Devlin et al., 2019; Radford et al., 2018), the models are now applied in domains such as audio (Agostinelli et al., 2023; Radford et al., 2023), images (Dosovitskiy et al., 2020; Kirillov et al., 2023), and video (Ravi et al., 2024). There is also growing interest in developing models that integrate different information modalities, such as text, images, and audio, or even depth or thermal data (Agrawal et al., 2024; Girdhar et al., 2023; Liu et al., 2025; OpenAI et al., 2023; Xu et al., 2025). Recently, we have seen a shift in model scaling from the pre-training phase to the post-training phase (El-Kishky et al., 2025; Guo et al., 2025), where reinforcement learning with verifiable rewards and methods to increase inference computation (test-time compute) (Akyürek et al., 2024; Snell et al., 2024) have shown their potential. This has led to new models with reasoning abilities, capable of tackling problems that were, until recently, reserved for human expertise (El-Kishky et al., 2025; Guo et al., 2025).

Scaling up these models requires increasingly powerful hardware resources, leading to an increase in training and inference costs. Although research is being conducted on model distillation to reduce the size of the set-ups (Guo et al., 2025), there is another solution inspired by Cajal’s observations on how different brain regions organize information in layers: Mixture of Experts (MoE) architectures. Originally conceived in the 1990s (Jacobs et al., 1991; Jordan and Jacobs, 1994), these architectures are now adopted by many leading language models. They include several ‘experts,’ each specializing in a specific knowledge area, along with a router or gate network that determines which expert processes the information (Fedus et al., 2022; Jiang et al., 2024; Liu et al., 2024). For instance, the recent multimodal model Llama 4 has two trillion parameters in total, but only 17 billion are activated at each inference.

## 6 Conclusion

The links between neuroscience and AI are evident, particularly in DL, which draws heavily on early discoveries regarding the brain’s structure and function. In the early days of AI, the two fields shared advances, and the boundaries between them were not clearly defined. Over time, however, the divide has become more pronounced, and many AI specialists work with little consideration for advances in neuroscience, despite their fundamental role in understanding intelligence and learning. Given that DL is currently one of the most promising approaches to achieving AGI and ASI, resuming collaboration between AI and neuroscience could offer new valuable perspectives. The work by Hassabis et al. (2017) provides an excellent overview of how neuroscience has inspired AI in the past, highlighting parallels with the present, and discussing future challenges that could be addressed jointly. Their conclusions make it clear that strengthening ties and synergies between the two fields would benefit both neuroscience and AI alike.

Although neuroscience played a key role the early development of ANNs, Cajal and Lorente de Nó received little to no recognition when the field first emerged. Their theories on the structure, function, and adaptability of neural circuits shaped our understanding of the brain and provided the conceptual groundwork for ANNs. Many of these ideas—already present in their early work of these two scientists—have endured and remain central to modern DL. The perceptron is a clear example: from Rosenblatt’s original recurrent formulation to its later feedforward version, it evolved into the multilayer perceptron, a backbone of today’s leading models, including transformers (see Figure 4). The fact that concepts first articulated by Cajal and Lorente de Nó—dynamic polarization, plasticity and recurrent circuits—remain central today underscores the foundational influence that these two pioneering neuroscientists have had on modern DL.

## Footnotes

^1^ https://ai.meta.com/blog/llama-4-multimodal-intelligence/
